# Treatment efficacy and re-infection rates of soil-transmitted helminths following mebendazole treatment in schoolchildren, Northwest Ethiopia

**DOI:** 10.1186/s41182-020-00282-z

**Published:** 2020-11-12

**Authors:** Ayalew Jejaw Zeleke, Abebe Genetu Bayih, Solomon Afework, John S. Gilleard

**Affiliations:** 1 Department of Medical Parasitology, School of Biomedical and Laboratory Sciences, College of Medicine and Health Sciences, University of Gondar, Gondar, Ethiopia; 2 Armauer Hansen Research Institute, Addis Ababa, Ethiopia; 3 Department of Internal Medicine, University of Gondar, Gondar, Ethiopia; 4 Department of Comparative Biology and Experimental Medicine, Faculty of Veterinary Medicine, Host-Parasite Interactions Program, University of Calgary, Calgary, Canada

**Keywords:** Soil-transmitted helminths, Efficacy, Re-infection, Schoolchildren, Mebendazole

## Abstract

**Background:**

Transmission of soil-transmitted helminth (STH) infection remains high in Ethiopia. This study aimed at assessing the therapeutic efficacy of mebendazole against soil-transmitted helminths and determining the re-infection rates of the parasites among schoolchildren in Northwest Ethiopia.

**Methods:**

A school-based cross-sectional study was conducted. Data was collected using a structured questionnaire. Stool specimens were examined using direct wet mount microscopy and Kato-Katz methods. Schoolchildren who tested positive for soil-transmitted helminths were treated with 500 mg single-dose of mebendazole. Cure and egg reduction rates were evaluated 2 to 3 weeks post treatment. Moreover, the re-infection rate of these parasites among those who were cured was determined 1 year after treatment. Data were analyzed using SPSS version 20. *P* value < 0.05 was considered as statistically significant.

**Result:**

A drug efficacy study was conducted on 62, 52, and 14 children infected by *Ascaris lumbricoides (A. lumbricoides)*, hookworm, and *Trichuris trichiura (T. trichiura)*, respectively. The cure rates (CR) of mebendazole against *A. lumbricoides*, hookworm, and *T. trichiura* were found to be 96.9%, 23.1%, and, 28.6%, respectively. The egg reduction rate (ERR) of *A. lumbricoides* was found to be 99.6% whereas 49.6% and 56.3% were reported for hookworm and *T. trichiura*, respectively. Eighty schoolchildren who were treated and cured from any STH infections were included for the determination of re-infection rate. Out of 80 children, 36.3% (29/80) were found to be re-infected after 1 year: 22 (75.9%), 6 (20.7%), and 1 (1.3%) of study participants were re-infected with *A. lumbricoides*, hookworm, and both infections, respectively. All re-infections were grouped under the “light infection” category.

**Conclusion:**

Mebendazole was found to be highly effective against *A. lumbricoides*, but had relatively low efficacy against hookworms and *T. trichiura*. These results bring into question the use of mebendazole in STH mass drug administration (MDA) programs in this region if albendazole, a drug with higher efficacy against hookworms, is available. Moreover, a significant number of treated children were re-infected with either or both of *A. lumbricoides* or hookworms 1 year after treatment emphasizing the need for better integrated intestinal helminthiasis control measures.

## Background

Soil-transmitted helminths are among the most widespread infectious agents in tropical and sub-tropical regions of the developing world. They are principally associated with inadequate sanitation, poverty, and poor water sources [1, 2]. Human intestinal helminthiasis is most commonly caused by soil-transmitted helminths (STHs), namely *Ascaris lumbricoides*, *Trichuris trichiura*, and hookworms (*Necator americanus* and *Ancylostoma duodenale)* [3].

Schoolchildren are high-risk groups for intestinal helminth infections. This has a significant adverse effect on survival, cognitive, and physical development of the children [4, 5]. Moreover, they are associated with poor school performance and absenteeism [6]. Although all STHs are associated with anemia, hookworm has a more pronounced effect on the child’s nutrition. It can also cause chronic intestinal blood loss which can result in iron deficiency anemia [5].

In Ethiopia, the current strategy to control STH infections is preventive chemotherapy, which involves a repeated large-scale administration of antihelmintic drugs to at-risk populations, most importantly to preschool and school-aged children. These mass drug administration (MDA) programs have been implemented to decrease the prevalence, intensity, and morbidity associated with STH infections. The MDA programs are implemented based on a periodic distribution of a single dose of a benzimidazole (BZ) drug (albendazole or mebendazole) [7]. Even though the anti-helmintic drugs are available and widely used, their efficacy varies and the drugs do not prevent re-infection. Moreover, low anthelminthic drug efficacy has been reported in helminth infections such as *T. trichiura* and hookworm*.* Similarly, other studies have highlighted the low cure rate (CR), e.g., 79.6% for hookworm and 63.1% for *T. trichiura* against mebendazole. In general, a number of studies have shown that the therapeutic efficacy of benzimidazole drugs against soil-transmitted helminths is decreased [8] (Ref).

The WHO (World Health Organization) recommends assessing the treatment efficacy, re-infection rates, and the effectiveness of MDA campaigns. This information is important for policy makers of a country about the value of MDA as a tool to eliminate morbidity caused by STHs [9]. Thus, this study aimed to assess the therapeutic efficacy of mebendazole against soil-transmitted helminths and determine the re-infection rates of the parasites among school children in Northwest Ethiopia.

## Materials and methods

### Study design and period

A school-based follow-up cross-sectional study was conducted. Study participants were recruited from Sanja (13° 20′ N/36° 45′ E), Maksegnit (12° 40′ N/37° 20′ E, Debark (13° 08′ N/37° 54′ E), Chuahit (12° 40′ N/37° 10′ E) Northwest Ethiopia. Base line data was collected from January 21 to February 21, 2018, while post treatment data was collected from February 4 to March 4, 2018. Moreover, the data for re-infection rate assessment was collected after a year within January 21 to 25, 2019.

### Sample size and sampling technique

The sample size was calculated based on WHO guidelines to assess the efficacy of antihelmintic drugs [10]. According to the guideline, a sample of 50 children positive for each of the parasites targeted by the investigators is sufficient to evaluate the efficacy of a drug. The number to be screened was estimated according to the prevalence in the study area and with the compliance rate of 80%. The prevalence of hookworm infection (*P* = 37.1%) was taken from a previous study conducted at Sanja town, northwest Ethiopia [11]. Therefore, the number of schoolchildren to be screened was calculated using the formula of number of expected study participants divided by compliance and prevalence rate of any of the STH infection. The final sample size was calculated to be a minimum of 504 schoolchildren. The sample size was equally divided amongst each site, i.e., 126 study participants from each study site. Schools and study participants were selected purposefully. Thus, a convenient sampling technique was used to recruit study participants in the study.

All children positive for any of the three soil-transmitted helminths (single infection), with a signed informed consent, and who did not have any additional health problems (based on medical history, physical examination, and vital signs) were included in the study. On the other hand, children who received any form of antihelmintic treatment within the past 30 days, had diarrhea, had experienced a severe allergic reaction to mebendazole, or were infected with other parasitic infections were excluded from the study.

### Baseline parasitological survey

Trained data collectors were recruited, and the data collection process was supervised by the investigators. At each data collection location, sufficient explanation about the aim of the research was given to the students, schoolteachers, and parents before the interview. Stool specimens were then collected from each schoolchild using a clean 10-g volume of stool sample container and labeled with unique identification number. The children were instructed on how to provide a stool specimen of their own, avoiding contamination with urine. Each stool sample was transported to the University of Gondar Laboratory within 2 h of collection. Each stool specimen was examined using wet mount microscopy and a Kato-Katz slide with 41.7 mg template size. The Kato-Katz slides were examined within 30–60 min of preparation for STH eggs. Then, infection intensity of all STHs was calculated by multiplying the average number of eggs counted in Kato-Katz slides by 24, which gives as the eggs per gram (epg) of stool. Results of the laboratory investigation were recorded on reporting sheets prepared for reporting of results. The infection intensities were classified as light, moderate, and heavy per the threshold set by WHO [12].

### Drug administration

Each child was given a light snack (e.g., a slice of bread or a biscuit) before the drug was administered. Then, each child was given a single dose of mebendazole (Vermox®) which was provided by the local coordinator office for the de-worming program. The expiry date and the storage condition of the drug were checked prior to administration. The tablet was administered by trained nurses, and each child was kept under direct observation for approximately 4 h. The children remained at school and continued their usual activities, but they were asked to report any side-effect to a member of the investigation team as soon as possible.

### Follow-up survey

Children who had a specimen positive for any STH infection and treated by mebendazole at baseline were requested to provide a second specimen within 14 to 21 days (2 to 3 weeks) from the treatment period. Schools were followed-up in the same order as in the baseline survey. Children who were absent at school on the follow-up day or did not bring a specimen were traced 1 or 2 days later. The laboratory methods used in the baseline survey were also applied in the follow-up survey [10].

### Re-infection rate assessment

Children who were positive for any STH infection during the baseline survey and negative following 14 to 21 days of post treatment were re-tested 12 months after the post-treatment period. The same laboratory techniques were utilized as in the baseline and post-treatment analysis.

### Quality assurance mechanisms

To avoid observer bias, two experienced microscopists performed the laboratory work. Independent readings of slides by the laboratory personnel were checked by another expert. The results of their observation were recorded for later comparison on separate sheets. A quality control was done by repeating all discordant results.

### Data management and analysis

Data was checked for completeness and then entered, edited, and cleaned using SPSS version 20 software. The overall mebendazole efficacy was determined and recorded using a descriptive method of data analysis. The following formula was used to calculate the cure rate (CR) and egg reduction rate (ERR) as it is described before [10].

$$ \mathrm{CR}=\frac{\mathrm{Number}\ \mathrm{of}\ \mathrm{negative}\ \mathrm{children}\ \mathrm{after}\ \mathrm{treatment}\ \mathrm{who}\ \mathrm{were}\ \mathrm{positive}\ \mathrm{at}\ \mathrm{baseline}\ }{\mathrm{numberofpositivechildrenbeforetreatment}}\ \mathrm{X}\ 100 $$

$$ \mathrm{ERR}=\left(1-\frac{\mathrm{Arithmetic}\ \mathrm{mean}\ \mathrm{egg}\ \mathrm{counts}\ \mathrm{at}\ \mathrm{follow}-\mathrm{up}\kern0.5em }{\mathrm{Arithmetic}\ \mathrm{mean}\ \mathrm{egg}\ \mathrm{counts}\ \mathrm{at}\ \mathrm{baseline}}\right)\mathrm{X}\ 100 $$

## Results

### Parasitological cure and egg reduction rates

A total of 130 schoolchildren who were infected with any STH species were treated with a 500-mg single dose of mebendazole. The number of schoolchildren who were infected with *A. lumbricoides*, hookworm, and *T. trichiura* were 64, 52, and 14, respectively. The cure rates of the drug against these parasites are shown in Table 1.

**Table 1:** Cure rates (CR) and 95% of CI of CR of mebendazole against STHs among schoolchildren in Northwest Ethiopia, 2018

Type of STHs	Cured, *n* (%), 95% CI	Uncured, *n* (%), 95% CI
*A. lumbricoides, n* = 64	62 (96.9), 92.6–101	2 (3.1), − 1–7
Hookworm, *n* = 52	12 (23.1), 11.6–34.5	40 (76.9), 65–88
*T. trichiura, n* = 14	4 (28.6), 4.9–52	10 (71.4), 47.7–95

*n* number of infected schoolchildren by each soil-transmitted helminths (STH), *CI* confidence interval

The ERR and mean egg intensities during pre-and post-treatment periods of the three soil-transmitted helminths are presented in Table 2. Moreover, their infection intensity category, both before and after treatment, is stated in the same table.

**Table 2:** STH infection intensity and egg reduction rates in pre- and post-treatment periods among schoolchildren in Northwest Ethiopia, 2018

Parasite species	Pretreatment, *n* (%)	Post treatment, *n* (%)	Egg reduction rate (%)
*A. lumbricoides, n = 64*			
Infection intensity			
Light	46 (71.9)	2 (100)	99.9
Moderate	17 (26.6)	0 (0)	
Heavy	1 (1.6)	0 (0)	
Mean epg	5186.4	2.8	
Hookworm, *n* = 52			
Infection intensity			
Light	52 (100)	40 (100)	49.6
Moderate	0 (0)	0 (0)	
Heavy	0 (0)	0 (0)	
Mean epg	334.4	168.5	
*T. trichiura*, *n* = 14			
Infection intensity			
Light	14 (100)	10 (100)	56.3
Moderate	0 (0)	0 (0)	
Heavy	0 (0)	0 (0)	
Mean epg	149	65.1	

*n* number of infected schoolchildren by each soil-transmitted helminths (STH)

### Re-infection rate of STH infections

Eighty schoolchildren who were treated and cured of any of the STH infections from 14 to 21 days post-treatment were included for the determination of the re-infection rate after 1 year. Accordingly, the STH re-infection rate was found to be 36.3% (29/80), and all were light infections. *A .lumbricoides* was the most frequently isolated parasite in the re-infection assessment study (Table 3).

**Table 3:** Intensity of STH infection among re-infected schoolchildren in Northwest Ethiopia, *n* = 80

	Re-infected, *n* (%)	Light	Moderate	Heavy	Mean epg
Al, *n* = 22	22 (75.9%)	22 (100%)	0 (0%)	0 (0%)	1054.7
Hw, *n* = 6	6 (20.7%)	7 (100%)	0 (0%)	0 (0%)	50.
AL + HW, *n* = 1	1 (3.4%)	1 (100)	–	–	–
Overall	29 (36.3)	–	–	–	–

*Al A. lumbricoides*, *Hw* Hookworm

Potential associations of the outcome variable with some common socio-demographic variables were examined. However, the re-infection rate did not show any significant association with age or sex (Table 4).

**Table 4:** Association of sex and age with re-infection rates of STH among schoolchildren in Northwest Ethiopia (*n* = 80)

Variables	Re-infection status		*P* value
	Re-infected, *n* (%)	Not re-infected, *n* (%)	
Sex			.364
Female	19 (38.8%)	30 (61.2%)	
Male	10 (32.3%)	21 (67.7%)	
Total	29 (36.3%)	41 (63.7%)	
Age in years			.558
7–12	22 (36.7%)	38 (63.3%)	
> 12	7 (35%)	13 (65%)	
Total	29 (36.3%)	41 (63.7%)	

## Discussion

Soil-transmitted helminths remain one of the most important public health problems in Ethiopia [13–15]. In the current study, the therapeutic efficacy of mebendazole against three soil-transmitted helminths has been evaluated among schoolchildren. Moreover, the children’s re-infection rate has been determined. According to this study, a 500-mg single dose of mebendazole had a satisfactory effect on *A. lumbricoides*. In other words, the CR against the parasite was found to be 96.9%. This is in line with studies conducted elsewhere in the world where soil-transmitted helminths are endemic (97.6%) [8], Western Indonesia (100%) [16], and Tanzania (95%) [17]. The second therapeutic efficacy indicator of antihelminthic drugs is egg reduction rate (ERR). Hence, the ERR of the same drug for *A .lumbricoides* treatment in this study was 99.9%. This is similar with the previous studies in other regions [8, 16, 17]. This study suggests that single dose of mebendazole is still highly effective for the treatment of ascariasis in Northwest Ethiopia.

Although the current study showed that mebendazole was very effective for ascariasis treatment, its cure rate for hookworms was only 23.1% and the ERR was only 49.6%. Similar findings have been reported from many different regions of the world e.g., cure rates in Tanzania (25%) [17], Southern Lao PDR (17.6%) [18] and Vietnam (38%) [19]. A review of 29 different efficacy studies found a mean cure rate of 25.5% (range 2.9–91.1%) for mebendazole against hookworms (Mrus 2018). Similarly, a meta-analysis of randomized controlled trials across many different countries (55 trials for cure rates and 46 trials for egg reduction rates) showed mebendazole to have much lower efficacy against hookworms than did albendazole: overall cure rates were 32.5% (95% confidence interval 20.8 to 46.9%) and 79.5% (95% confidence interval 71.5 to 85.6%) and overall egg reduction rates were 61.0% (95% confidence interval 52.0 to 69.9%) and 89.6% (95%, 81.9 to 97.3%) for mebendazole and albendazole, respectively [18, 19] (Ref). This study further indicates that a single dose of mebendazole is not very effective for hookworm treatment in Northern Ethiopia and that its use as part of MDA programs in the region should be re-evaluated.

As far as the efficacy of mebendazole against *T. trichuira* is concerned, its cure rate was unsatisfactory being only 28.6%. The ERR was also found to be low (56.3%). This finding is similar to studies conducted in Tanzania (value) [17] and Southern Lao PDR (17.6%) [18]. Hence, this study indicates that mebendazole has low efficacy against both hookworm and *T. trichuira*. As with other studies, it is unclear whether this reflects some degree of anthelmintic drug resistance and further work will be required to investigate the reason behind this observation.

The current study has also assessed the re-infection status of the schoolchildren due to soil-transmitted helminthiasis after a year of cure time. Accordingly, a re-infection rate of 36.3% (95% CI, 30–53%) was observed. Similar re-infection findings were reported from Chencha District, southern Ethiopia (36.8%) [20]. The re-infection rate was mainly due to *A. lumbricoides* (75.9%) followed by hookworm (20.9%). The higher re-infection rate by *A. lumbricoides* than the other two STH infections might be related with its ubiquitous nature and large levels of environmental contamination due to the extreme resistance of its egg to harsh environments. The incidence of a significant number of re-infections by STHs in the region may partly indicate that the existing prevention and control strategies are not adequate enough for the achievement of elimination goals of soil-transmitted helminthiasis related morbidity in the country by 2020 [21].

Finally, the present study revealed that STH re-infection rate among schoolchildren did not show any statistically significant association with sex or age. This implies that the probability of exposure for all schoolchildren in the study area was comparable. Thus, further comprehensive studies which include the description of several possible factors leading to frequent infections of schoolchildren by STHs are necessary in the area.

The limitation of this study is that only a single stool specimen had been used for parasite investigation which might have affected the detection rate of the parasite due to intermittent egg excretion rates by the adult parasites. Moreover, only 14 study participants were involved for *T. trichiura* drug efficacy study and this might have also affected the interpretation of mebendazole efficacy against *T. trichiura*.

## Conclusion

Mebendazole was found to be highly effective against *A. lumbricoides.* However, its therapeutic efficacy was low against both hookworm and *T. trichiura*. This questions its use in MDA programs if albendazole, a more efficacious drug against hookworms, is available. Moreover, a considerable number of re-infection rates within a year of the post-treatment period may indicate that the elimination and control of soil-transmitted helminths is challenged by several factors. Thus, the implementation of more evidence-based and integrated preventive strategies is an important goal.

## Data Availability

All data generated or analyzed during this study are included in this published article.
